# Short-Term Limited Duration Insurance Plan Policies and Cancer Stage at Diagnosis

**DOI:** 10.1001/jamanetworkopen.2025.1075

**Published:** 2025-03-18

**Authors:** Nuo Nova Yang, Jingxuan Zhao, Justin M. Barnes, Anne C. Kirchhoff, Fumiko Chino, K. Robin Yabroff, Xuesong Han

**Affiliations:** 1 Surveillance and Health Equity Science, American Cancer Society, Atlanta, Georgia; 2 Department of Radiation Oncology, Washington University School of Medicine in St Louis, St Louis, Missouri; 3 Department of Pediatrics, University of Utah School of Medicine, Salt Lake City; 4 Huntsman Cancer Institute, Salt Lake City, Utah; 5 The University of Texas MD Anderson Cancer Center, Houston

## Abstract

**Importance:**

Many short-term limited duration (STLD) insurance plans do not cover essential health benefits, such as cancer screenings, potentially delaying diagnoses. In October 2018, federal regulations loosened restrictions on STLD plans by extending the allowable duration from 3 months to 36 months. States expanded, restricted, or eliminated the sale or terms of STLD plans, both prior to 2018 and afterwards. The association of state-level policies and late-stage cancer diagnosis is largely undocumented.

**Objective:**

To examine associations of state-level policies regarding STLD plans and late-stage cancer diagnosis before and after the 2018 federal rule.

**Design, Setting, and Participants:**

This cross-sectional study included adults aged 18 to 64 years newly diagnosed with cancer (January 2016 to February 2020) in 47 states and the District of Columbia from the National Cancer Database. Patients were grouped by state-level STLD plan policies: (1) states continuously prohibited STLD plans before and after 2018; (2) states stopped offering STLD plans after 2018; (3) states allowed the sale of STLD plans with additional restrictions to the 2018 federal rules; (4) states with no additional STLD regulation. Difference-in-differences analyses were conducted to evaluate changes in late-stage diagnoses (stages III/IV) pre-2018 (January 2016 to June 2018) and post-2018 (January 2019 to February 2020) for groups 2, 3, and 4 vs group 1 for all cancers combined and for 5 common cancers, adjusting for sociodemographic factors (age group, sex, metropolitan status, zip code-level poverty), diagnosis year, and state random effect. All analyses were performed from May 2023 to January 2025.

**Exposures:**

State-level STLD plan policies.

**Main Outcomes and Measures:**

Change in percentages of late-stage cancer diagnosis.

**Results:**

Among 1 289 366 adults newly diagnosed with cancer (mean [SD] age, 53 [9.2] years; 536 514 [41.6%] female; 73 061 [5.7%] Hispanic, 110 564 [8.6%] non-Hispanic Asian and Pacific Islander and other, 172 246 [13.4%] non-Hispanic Black, 921 722 [71.5%] non-Hispanic White), 604 882 (46.9%) were from group 4 states (no additional STLD regulations). Group 4 had a net increase of 0.76 (95% CI, 0.22-1.29) percentage points (*P* = .01) and group 3 (some STLD regulations) had a net increase of 0.84 (95% CI, 0.26-1.42) percentage points (*P* = .005) in late-stage diagnoses compared with group 1 (continuously prohibited STLD plans). Similar patterns were observed for female breast and colorectal cancers.

**Conclusions and Relevance:**

In this cross-sectional study of state-level policies regarding STLD plans and late-stage cancer diagnosis, loosened federal restrictions on STLD plans in 2018 were associated with a net increase in late-stage cancer diagnoses in states without or with inadequate regulatory protections, underscoring the critical role of state policies in regulating STLD plans.

## Key Points

**Question:**

Is there an association of federal and state policies regulating short-term limited duration (STLD) insurance plans with cancer diagnosis stage?

**Findings:**

In this cross-sectional study of 1 289 366 adults aged 18 to 64 years newly diagnosed with cancer from January 2016 to February 2020, patients in states with no or some STLD regulations had net increases of 0.76 and 0.84 percentage points in late-stage diagnoses, respectively, following the 2018 federal rule loosening restrictions of STLD plans compared with states that continuously prohibited STLD plans.

**Meaning:**

The 2018 federal rule loosening restrictions on STLD plans was associated with increased late-stage cancer diagnoses in states without or with inadequate regulatory protections.

## Teaser

This cross-sectional study investigates state-level policies regarding short-term limited duration insurance plans and late-stage cancer diagnosis before and after a 2018 federal rule loosening restrictions on the duration of these plans.

## Introduction

Short-term limited duration (STLD) plans are a type of coverage designed to fill temporary insurance gaps that may occur during employment transitions or other coverage changes.^1^ STLD plans do not have the consumer protections and comprehensive coverage required by the Patient Protection and Affordable Care Act (ACA). Many STLD plans do not cover essential health benefits, such as cancer screenings, thus may result in delayed cancer diagnoses and increased disease burden once diagnosed. STLD plans also have high patient cost-sharing (ie, high deductibles, no or high annual out-of-pocket maximum) for the benefits they do cover. Even if cancer screening is covered, STLD plans can impose cost-sharing for workup of new cancer-associated symptoms that would otherwise have no out-of-pocket costs under ACA-compliant plans. Enrolling in an STLD plan has been estimated to cost a newly diagnosed patient with lymphoma $16 800 more in out-of-pocket expenses than an ACA-compliant plan.^2^ STLD plans may also weaken the ACA-compliant health insurance market through adverse selection, as healthier people may choose STLD plans because of lower premiums and leave the ACA-compliant plans a pool of people with higher health care needs.^3^

Patients with cancer diagnosed at later stages face less effective treatments and financial risk, elevating the risk of poor cancer outcomes.^4^ Additional barriers faced by patients diagnosed with cancer while on a STLD plan include coverage denial, delays in treatment, and high out-of-pocket costs, which can adversely affect survival.^5,6^

Prior to 2018, allowable duration of STLD plans under federal rules was 3 months in all states. In October 2018, a federal rule loosened restrictions to less than 12 months with the option to renew for a total duration up to 36 months.^7^ States have the option to impose more stringent regulations to restrict or ban STLD plans. Some individual states opted to restrict or eliminate the sale or terms of STLD plans, both prior to 2018 and afterwards.^8^ As of February 2020, 5 states that prohibited STLD plans before the 2018 federal rule continued prohibiting such plans; 6 states stopped offering STLD plans after the 2018 rule; 5 states kept the same 3-month limit on STLD plans before and after the 2018 federal rule; 12 states imposed more stringent regulations on STLD plans in addition to the 2018 federal rule (eg, limited STLD plans to 6 months in duration and prohibiting renewals); and 23 states followed the federal rule with no additional regulation (Figure) Following the 2018 rule, enrollment in STLD plans grew 27% from approximately 2.36 million individuals in 2018 to 3 million in 2019.^9^ In this study, we examined associations of state policies regulating STLD plans and late-stage cancer diagnosis before and after the 2018 federal rule, using nationwide hospital-based cancer registry data.

**Figure.**
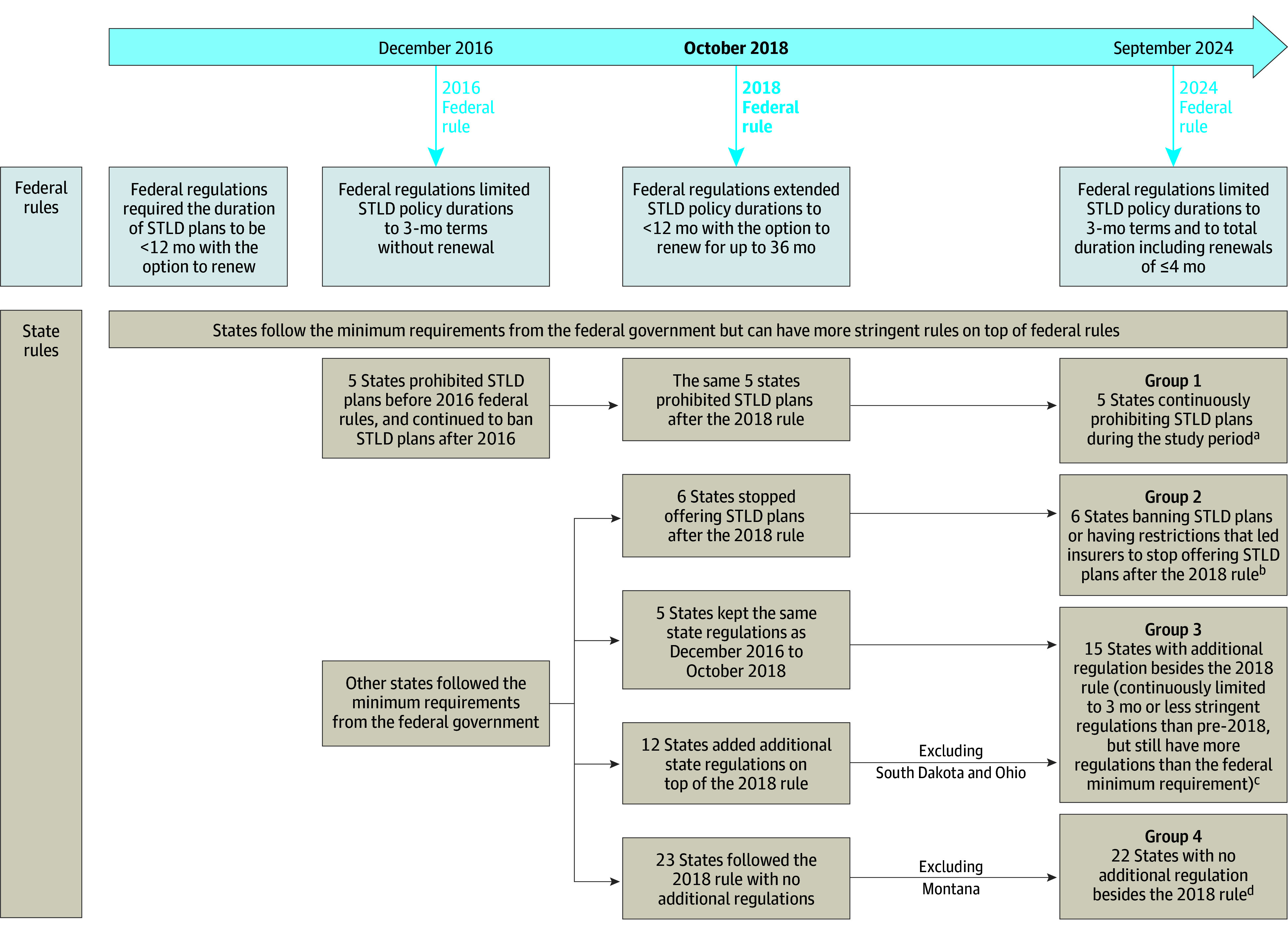
Short-Term Limited Duration (STLD) Insurance Plan Policies Before and After October 2018 Federal Rule ^a^Group 1 included states continuously prohibiting STLD plans during the study period: New York, New Jersey, Massachusetts, Rhode Island, and Vermont. ^b^Group 2 included states banning STLD plans or having restrictions that led insurers to stop offering STLD plans after the 2018 federal rule: California, Connecticut, Hawaii, New Mexico, Colorado, and Maine. ^c^Group 3 included states with some additional regulation besides the 2018 federal rule: District of Columbia, Delaware, Illinois, Kansas, Maryland, Michigan, Minnesota, Missouri, North Dakota, New Hampshire, Nevada, Oregon, South Carolina, Washington, and Wisconsin. ^d^Group 4 included states with no additional regulation besides the 2018 federal rule: Alabama, Alaska, Arkansas, Arizona, Florida, Georgia, Iowa, Idaho, Indiana, Kentucky, Louisiana, Mississippi, Nebraska, North Carolina, Oklahoma, Pennsylvania, Tennessee, Utah, Texas, Virginia, West Virginia, and Wyoming.

## Methods

This cross-sectional study was exempted by the Morehouse School of Medicine institutional review board and informed consent was not required because the data are deidentified. The results reporting followed the Strengthening the Reporting of Observational Studies in Epidemiology (STROBE) reporting guideline.

We used data from the National Cancer Database (NCDB), a hospital-based cancer registry that captures approximately 72% of all newly diagnosed cancer cases in the US.^10^ We identified adults aged 18 to 64 years diagnosed with a stageable cancer between January 1, 2016, and February 29, 2020, from 47 states and the District of Columbia; adults diagnosed in 3 states with discrepancies in their state-level policies and the availability of STLD plans were excluded (eFigure 1 in Supplement 1). Sociodemographic information in the NCDB, including race and ethnicity, was routinely collected by the cancer registrars through reviewing medical records. Adults diagnosed after February 2020 were excluded because of disruptions in health care due to the COVID-19 pandemic.

Our primary outcome was percentage of late-stage cancer diagnosis (stages III/IV). In this quasi-experimental study, patients were categorized into 4 groups according to their states’ STLD policies and availability as of February 2020: (1) states continuously prohibiting STLD plans during the study period as the control group; and the following 3 treatment groups: (2) states with no restrictions beyond the federal rule prior to 2018 but banning STLD plans or having restrictions that led insurers to stop offering STLD plans after the 2018 federal rule; (3) states that had restrictions in addition to the 2018 federal rule, including states that kept the same state regulation that were already more stringent than the 2018 federal minimum, and states that added additional regulations on top of the 2018 rule; and (4) states that had no additional regulation of STLD plans after the 2018 federal rule (Figure; eFigure 2 in Supplement 1).

### Statistical Analysis

After examining sample characteristics by state groups, we conducted difference-in-differences (DiD) analyses to evaluate changes in percentages of late-stage diagnoses (stages III/IV) before (January 2016 to June 2018) and after (January 2019 to February 2020) federal STLD plan expansion between groups 2 (banned after 2018), 3 (some STLD regulations), and 4 (no additional STLD regulations) vs group 1 (continuously prohibited STLD plans) for all cancers combined and for 5 common cancers with either effective screening tests or early signs and symptoms: female breast cancer, prostate cancer, colorectal cancer, non–small-cell lung cancer, and melanoma. Although screening for cervical cancer is effective, relatively small numbers of late-stage disease limited further evaluation. The DiD design can account for changes in health policy and American Joint Committee on Cancer (AJCC) stage system that affect all groups equally. We fitted linear probability models and adjusted for age group, sex, metropolitan status, zip code–level poverty as confounders that indicate patients’ demographic and socioeconomic status and potentially affect patients access to health care. We also adjusted for diagnosis year to account for general temporal trend, and state random effect to account for other state-specific variations, such as state-level changes in health or other policies, that could influence cancer stage at diagnosis. Parallel trend assumption for the DiD model was assessed by testing the statistical significance of a state policy group-by-quarter interaction term before policy implementation (eTable 1 in Supplement 1). Sensitivity analyses restricted the sample to recommend ages for screening (ie, 40-64 years for female breast cancer and 50-64 years for colorectal cancer) (eTable 2 in Supplement 1). Additional sensitivity analysis further adjusted for zip-code level education attainment and state-level uninsured rate (eTable 4 in Supplement 1). Supplemental analysis examined stage among adults aged 66 to 70 years, who would be minimally affected by STLD policies, as a falsification test (eTable 3 in Supplement 1). Two-sided *P* < .05 was considered statistically significant. We also calculated 99% CIs for the main analysis estimates considering the large sample size (eTable 5 in Supplement 1). Statistical analysis was performed from May 2023 to January 2025 using SAS version 9.4 (SAS Institute).

## Results

A total of 1 289 366 adults aged 18 to 64 years newly diagnosed with cancer were included; 536 514 (41.6%) were female; 73 061 (5.7%) were Hispanic; 110 564 [8.6%] were non-Hispanic Asian and Pacific Islander and other, 172 246 (13.4%) were non-Hispanic Black, 921 722 (71.5%) were non-Hispanic White; 604 882 (46.9%) were from group 4 states (no additional STLD regulations); and the mean (SD) age of the sample at diagnosis was 53 (9.2) years. There were demographic variations among the 4 groups of states, partly reflecting their geographic distribution. For example, group 1 (continuously prohibited STLD plans) had the highest percentage of Hispanic and non-Hispanic Asian and Pacific Islander and other patients, group 4 (no additional STLD regulations) had the highest percentage of non-Hispanic Black patients, and group 3 (some STLD regulations) had the highest percentage of non-Hispanic White patients. Area-level median income was highest in group 1 (continuously prohibited STLD plans) and lowest in group 4 (no additional STLD regulations) (Table 1).

**Table 1.** Characteristics of Newly Diagnosed Patients With Cancer by States’ STLD Plan Policy^a^

Characteristic	Patients, No. (%) (N = 1 289 366)			
	Group 1: States prohibiting STLD plans prior to 2016	Group 2: States banning STLD plans or having strict regulations after 2018	Group 3: States restricting federal allowed STLD plans	Group 4: States with no STLD regulation
Overall age, mean (SD), y	53 (9.2)	53 (9.4)	54 (9.2)	53 (9.2)
Overall^b^	175 322 (13.6)	174 080 (13.5)	335 082 (26.0)	604 882 (46.9)
Year of diagnosis				
Pre-STLD rule (Jan 2016 to June 2018)	119 949 (68.4)	119 352 (68.6)	231 381 (69.1)	414 504 (68.5)
Post-STLD rule (Jan 2019 to Feb 2020)	55 373 (31.6)	54 728 (31.4)	103 701 (31.0)	190 378 (31.5)
Sex				
Female	103 777 (59.2)	106 160 (61.0)	193 295 (57.7)	349 620 (57.8)
Male	71 545 (40.8)	67 920 (39.0)	141 787 (42.3)	255 262 (42.2)
Race and ethnicity				
Hispanic	14 461 (8.3)	24 040 (13.8)	14 334 (4.3)	20 226 (3.3)
Non-Hispanic Asian and Pacific Islander and other^c^	16 617 (9.5)	32 281 (18.5)	13 912 (4.2)	47 754 (7.9)
Non-Hispanic Black	20 947 (12.0)	10 717 (6.2)	43 100 (12.9)	97 482 (16.1)
Non-Hispanic White	120 286 (68.6)	105 673 (60.7)	261 010 (77.9)	434 753 (71.9)
Unknown	3011 (1.7)	1369 (0.8)	2726 (0.8)	4667 (0.8)
Age group, y				
18-39	16 866 (9.6)	18 386 (10.6)	31 334 (9.4)	57 748 (9.6)
40-49	31 063 (17.7)	32 444 (18.6)	57 247 (17.1)	107 427 (17.8)
50-59	74 377 (42.4)	71 379 (41.0)	140 778 (42.0)	254 406 (42.1)
60-64	53 016 (30.2)	51 871 (29.8)	105 723 (31.6)	185 301 (30.6)
Rural-urban status				
Metro area with population ≥1 000 000	133 249 (76.0)	97 974 (56.3)	181 413 (54.1)	281 962 (46.6)
Metro area with population <1 000 000	26 497 (15.1)	60 681 (34.9)	92 929 (27.7)	201 126 (33.3)
Nonmetro area	8377 (4.8)	11 895 (6.8)	45 946 (13.7)	99 301 (16.4)
Unknown	7199 (4.1)	3530 (2.0)	14 794 (4.4)	22 493 (3.7)
Zip code–level poverty, %				
<5.0	46 574 (26.6)	24 704 (14.2)	49 272 (14.7)	60 105 (9.9)
5.0-9.9	53 606 (30.6)	51 643 (29.7)	97 675 (29.2)	131 437 (21.7)
10.0-19.9	46 192 (26.4)	62 728 (36.0)	126 750 (37.8)	255 314 (42.2)
>20.0	28 675 (16.4)	34 614 (19.9)	60 613 (18.1)	157 096 (26.0)
Unknown	275 (0.2)	391 (0.2)	772 (0.2)	930 (0.2)
Cancer site				
Female breast	45 866 (26.2)	47 825 (27.5)	83 909 (25.0)	150 077 (24.8)
Prostate	23 323 (13.3)	19 865 (11.4)	44 985 (13.4)	75 054 (12.4)
Colorectal	16 573 (9.5)	18 753 (10.8)	34 017 (10.2)	64 453 (10.7)
Non–small cell lung	15 085 (8.6)	11 331 (6.5)	29 627 (8.8)	56 796 (9.4)
Uterine	9572 (5.5)	10 848 (6.2)	18 850 (5.6)	32 528 (5.4)
Melanoma	7063 (4.0)	8543 (4.9)	17 744 (5.3)	28 245 (4.7)
Kidney	8364 (4.8)	8716 (5.0)	17 111 (5.1)	33 426 (5.5)
Cervical	2970 (1.7)	3699 (2.1)	5946 (1.8)	13 188 (2.2)
Pancreas	4914 (2.8)	4585 (2.6)	9198 (2.74)	16 653 (2.8)
Other^d^	41 592 (23.7)	39 915 (22.9)	73 695 (22.0)	134 462 (22.2)
Facility type				
Community cancer program	6738 (3.8)	8292 (4.8)	14 427 (4.3)	31 500 (5.2)
Comprehensive community cancer program	35 123 (20.0)	66 028 (37.9)	100 513 (30.0)	222 693 (36.8)
Academic comprehensive cancer program	62 268 (35.5)	32 463 (18.7)	70 141 (20.9)	137 165 (22.7)
NCI-designated comprehensive cancer center program	45 127 (25.7)	30 075 (17.3)	53 997 (16.1)	92 296 (15.3)
Other	26 066 (14.9)	37 222 (21.4)	96 004 (28.7)	121 228 (20.0)

Abbreviations: metro, metropolitan; NCI, National Cancer Institute; STLD, short-term limited duration insurance plan.

^a^ Data source: National Cancer Database 2016-2020.

^b^ Overall percentage is presented as row percentage, the rest of the table shows column percentage.

^c^ Other included non-Hispanic American Indian and Alaska Native and other ethnicity groups.

^d^ Other included all other stageable cancer.

The percentages of late-stage diagnosis decreased after 2018 in all state groups (Table 2). Compared with patients in group 1 states (continuously prohibited STLD plans), group 4 states (no additional STLD regulations) had a net increase of 0.76 (95% CI, 0.22 to 1.29) percentage points (*P* = .01) in late-stage diagnosis, group 3 states (some STLD regulations) had a net increase of 0.84 (95% CI, 0.26 to 1.42) percentage points (*P* = .005), and group 2 states (stopped offering STLD plans since 2018) had no significant change of 0.45 (95% CI, −0.22 to 1.12) percentage points (*P* = .19) in late-stage diagnoses (Table 2).

**Table 2.** Association of State Short-Term Limited Duration Plan Policy and Percentage of Late-Stage Cancer Diagnosis^a^

Characteristic	No.	Late-stage diagnosis, %		Absolute difference, percentage points (95% CI)	Unadjusted model		Adjusted model	
		2016-2018	2019-2020		Difference-in-differences (95% CI)	*P* value	Difference-in-differences (95% CI)	*P* value
**All cancer**								
Group 1	175 322	33.77	32.11	−1.66 (−2.14 to −1.18)	1 [Reference]	NA	1 [Reference]	NA
Group 2	174 080	34.09	32.65	−1.44 (−1.92 to −0.95)	0.22 (−0.46 to 0.91)	.52	0.45 (−0.22 to 1.12)	.19
Group 3	335 082	35.40	34.36	−1.04 (−1.39 to −0.69)	0.62 (0.02 to 1.22)	.04	0.84 (0.26 to 1.42)	.005
Group 4	604 882	37.11	36.03	−1.08 (−1.34 to −0.82)	0.58 (0.03 to 1.13)	.04	0.76 (0.22 to 1.29)	.01
**Female breast**								
Group 1	45 866	13.47	11.99	−1.48 (−2.17 to −0.79)	1 [Reference]	NA	1 [Reference]	NA
Group 2	47 825	14.76	14.07	−0.68 (−1.36 to −0.01)	0.79 (−0.17 to 1.76)	.11	0.83 (−0.13 to 1.79)	.09
Group 3	83 909	14.33	13.51	−0.83 (−1.34 to −0.31)	0.65 (−0.21 to 1.51)	.14	0.73 (−0.12 to 1.59)	.09
Group 4	150 077	15.71	15.12	−0.59 (−0.98 to −0.21)	0.89 (0.09 to 1.68)	.03	1.01 (0.22 to 1.79)	.01
**Prostate**								
Group 1	23 323	27.40	30.70	3.31 (2.08 to 4.55)	1 [Reference]	NA	1 [Reference]	NA
Group 2	19 865	29.26	33.46	4.20 (2.86 to 5.54)	0.88 (−0.94 to 2.70)	.34	0.99 (−0.83 to 2.80)	.29
Group 3	44 985	27.12	33.21	6.09 (5.19 to 6.99)	2.77 (1.25 to 4.30)	<.001	2.88 (1.36 to 4.40)	<.001
Group 4	75 054	26.78	33.34	6.56 (5.87 to 7.25)	3.25 (1.83 to 4.66)	<.001	3.27 (1.86 to 4.68)	<.001
**Colorectal**								
Group 1	16 573	55.57	58.29	2.72 (1.11 to 4.33)	1 [Reference]	NA	1 [Reference]	NA
Group 2	18 753	53.87	58.35	4.48 (2.95 to 6.00)	1.75 (−0.46 to 3.97)	.12	1.68 (−0.52 to 3.89)	.13
Group 3	34 017	54.44	59.77	5.33 (4.20 to 6.47)	2.61 (0.64 to 4.58)	.01	2.55 (0.59 to 4.52)	.01
Group 4	64 453	57.16	60.74	3.58 (2.75 to 4.40)	0.86 (−0.95 to 2.66)	.35	0.72 (−1.08 to 2.52)	.43
**Non–small cell lung**								
Group 1	15 085	63.38	61.67	−1.72 (−3.34 to −0.09)	1 [Reference]	NA	1 [Reference]	NA
Group 2	11 331	69.37	68.89	−0.48 (−2.38 to 1.41)	1.23 (−1.26 to 3.73)	.33	1.15 (−1.32 to 3.62)	.36
Group 3	29 627	67.05	65.54	−1.51 (−2.67 to −0.34)	0.21 (−1.79 to 2.21)	.84	0.31 (−1.67 to 2.29)	.76
Group 4	56 796	68.78	67.40	−1.38 (−2.22 to −0.54)	0.34 (−1.49 to 2.17)	.72	0.51 (−1.30 to 2.32)	.58
**Melanoma**								
Group 1	7063	17.26	17.72	0.46 (−1.57 to 2.49)	1 [Reference]	NA	1 [Reference]	NA
Group 2	8543	18.73	20.62	1.89 (0.03 to 3.75)	1.43 (−1.32 to 4.18)	.31	1.46 (−1.26 to 4.19)	.29
Group 3	17 744	18.85	19.09	0.24 (−1.03 to 1.51)	−0.22 (−2.62 to 2.17)	.85	−0.13 (−2.50 to 2.24)	.91
Group 4	28 245	21.05	22.12	1.07 (0.07 to 2.07)	0.61 (−1.65 to 2.87)	.60	0.55 (−1.69 to 2.79)	.63

Abbreviation: NA, not applicable.

^a^ Data source: National Cancer Database 2016-2020.

When stratified by cancer site, there was a significant increase in late-stage diagnosis in group 3 (some STLD regulations) relative to group 1 states (continuously prohibited STLD plans) for colorectal (2.55 [95% CI, 0.59-4.52] percentage points; *P* = .01) and prostate (2.88 [95% CI, 1.36-4.40] percentage points; *P* < .001) cancers. There was a significant increase in late-stage diagnosis in group 4 (no additional STLD regulations) relative to group 1 states (continuously prohibited STLD plans) for female breast (1.01 [95% CI, 0.22-1.79] percentage points; *P* = .01) and prostate (3.27 [95% CI, 1.86-4.68]; *P* < .001) cancers.

We found similar patterns in female breast cancer and colorectal cancer when restricting sample to recommend screening ages (eTable 2 in Supplement 1). In the falsification test conducted among older adults aged 66 to 70 years, differences by state policy groups were generally statistically nonsignificant (eTable 3 in Supplement 1). Our sensitivity analysis results yielded similar results after including zip code–level education attainment and state-level uninsured rate as confounders (eTable 5 in Supplement 1).

## Discussion

This large national quasi-experimental cross-sectional study found that the 2018 federal policy loosening restrictions on STLD plans was associated with an increase in late-stage cancer diagnoses in states without or with inadequate additional STLD plan regulatory protections. Findings were consistent among cancer types with recommend screening tests (ie, female breast and colorectal cancers) and extended prior research conducted in a limited number of states, underscoring the importance of state policies and federal efforts to limit STLD plans.^11,12^ Considerable overlap exists between states lacking or having inadequate state-level STLD plan protections and states with large rural populations in the southern region of the US, which are states that already experience some of the highest cancer mortality rates in the country.^13^ Thus, lack of federal and state STLD plan protections have the potential to widen existing geographic disparities in cancer outcomes. On the other hand, the declines in percentages of late-stage diagnosis across all STLD groups after 2018 reflects the overall advancements in cancer early diagnosis.

Additionally, STLD plans can disrupt the individual health insurance market, which provides insurance coverage for about 10% of people aged 18 to 64 years.^14^ Premiums in states that allowed proliferation of STLD plans increased by 4.3%; whereas premiums in states with restrictions decreased by 1% in 2020.^2^ Future research is needed to evaluate whether STLD plans may contribute to higher premiums in the ACA-compliant individual market when younger and healthier enrollees opt for cheaper plans which adversely affect the risk pool for ACA-compliant plans.^2^ In our sensitivity analysis, adjusting for education attainment and state-level uninsured rate showed similar results. Our results align with the findings from a previous study, which showed that tightening regulations on STLD plans are unlikely to have substantial negative effects on uninsured rate.^15^

By May 2024, STLD plans had become unavailable in 3 more states (New Hampshire, Minnesota, Washington) and the District of Columbia.^8^ Our study has important policy implications given that the 2024 rule to limit the use of STLD to 3 months, and to total duration—including renewals—of no more than 4 months. This new final rule applies to STLD plans beginning on or after September 1, 2024.^12^ Federal rules supersede state rules if federal rules are more stringent, therefore more states would be further limited by the new federal rule. However, STLD plans that were already in effect, or policies that were sold and issued before September 1, 2024, are not impacted by the new rule and will continue to comply with previous federal and state rules. It is up to the state and insurers whether an STLD plan is available for sale in that state. The incoming administration may again revert to loosening restrictions on STLD plans. Alternatively, for individuals who need short-term coverage, marketplace options that cover the ACA’s essential benefits are available and are offered on a month-to-month basis. An estimation with the 2024 Marketplace open enrollment data showed that advance premium tax credits are available for about 92% of eligible individuals and will reduce the mean monthly premiums to $74 per month.^16,17^

### Limitations

This study has limitations. Although the NCDB includes 72% of newly diagnosed cancer cases, it is hospital-based rather than population-based. Information about private insurance plan type among newly diagnosed patients was unavailable from the NCDB and as a result we did not have information on whether or not individuals had coverage from STLD plans in states that allowed them. We included all individuals with the selected cancer types because STLD plans may affect enrollees’ access to any care, therefore delaying care to evaluate early signs and symptoms and limiting early detection, given the non-ACA-compliant nature of such plans. Another limitation is the coverage of melanoma (52%) in NCDB is relatively low compared with other cancer sites (>70%).^18^ We included melanoma because melanoma diagnosis reflects access to care through early detection of symptoms. The association of STLD plans and late-stage melanoma diagnosis should be interpreted with caution given the lower coverage in NCDB.

Despite the use of a rigorous quasi-experimental design, we reported associations of state policies and cancer stage, rather than causality, in this cross-sectional study. Differential policy climate and implementation of other cancer screening and early detection programs, including the National Breast and Cervical Early Detection Program (NBCCEDP), may also impact state level differences in late-stage cancer diagnoses. However, because there is limited data about receipt of screening from these programs, accurate state-level model adjustment was not possible.^19^ While we accounted for state-specific variation that could affect cancer diagnosis stage by including a state random effect in multivariable models, there were various other health and public policies enacted over the study period that could affect insurance coverage and/or access to care that could also influence our findings.^11^ Similarly, states may vary in implementation and enforcement of STLD plans; these data are also unavailable. Also, the publication of the AJCC 8th edition, in 2016 and its implementation in 2018 could affect cancer stage at diagnosis during our study period.^20^ However, we included year of diagnosis to account for the temporal trend, and the DiD approach effectively removes the influences of time-varying factors during the study period that affect all groups equally. Additionally, although we included available measures of socio-demographics, we could not account for unmeasured potential confounders, such as individual-level income and employment status, which could be associated with state STLD policies and diagnosis stage.

To fully assess the impact of STLD plans on cancer diagnoses remains challenging because there is limited information about the total number of STLD plans in effect and the number of enrollees in the US.^5^ Health provider organizations can sell STLD plans through associations and non-group market or file their plans under other categories of coverage with insurance departments, both of which make reporting the true enrollment of STLD plans difficult.^5^ More institutional efforts are needed to require reporting of enrollment from STLD plan sellers. Future cohort studies with individual-level STLD plan enrollment information and follow-up for cancer outcomes are warranted to confirm the delaying effects of STLD on cancer diagnosis. Meanwhile, regulating the marketing of STLD plans in states where STLD plans are still available is important for halting the spread of misinformation and increasing public awareness.

## Conclusions

In this large national cross-sectional study of federal and state-level policies regarding STLD plans and late-stage cancer diagnosis, the federal rule loosening restrictions on STLD plans was associated with a net increase in late-stage cancer diagnoses in states without or with inadequate regulatory protections. Future studies to monitor the federal and state STLD policies regarding cancer care and outcomes are warranted.

## Notes

Supplement 1. **eFigure 1.** Study Sample **eFigure 2.** STLD Policy Status by States as of February 29th, 2020 **eTable 1.** Parallel Trend Assumption Test **eTable 2.** Association of State STLD Plan Policies and Later-Stage Cancer Diagnoses: Sensitivity Analysis for Female Breast Cancer and Colorectal Cancer in Age Group with Recommended Asymptomatic Screening **eTable 3.** Association of State Short-Term Limited Duration Plan Policies and Later-Stage Cancer Diagnoses: Falsification Test Among Adults Aged 66-70 Years Age Eligible for Medicare Coverage **eTable 4.** Association of State STLD Plan Policies and Later-Stage Cancer Diagnoses: Sensitivity Analysis Adjusting for Zip-Code Education Attainment and State-Level Uninsured Rate **eTable 5.** Association of State STLD Plan Policies and Later-Stage Cancer Diagnoses With 99% Confidence Interval

Supplement 2.
Data Sharing Statement
