# Supplementary material for: Short-Term Limited Duration Insurance Plan Policies and Cancer Stage at Diagnosis
Source: JAMA Netw Open. 2025 Mar 18;8(3):e251075. doi: 10.1001/jamanetworkopen.2025.1075 (PMC11920836; doi:10.1001/jamanetworkopen.2025.1075)
Supplement: Supplement 1. — eFigure 1. Study Sample eFigure 2. STLD Policy Status by States as of February 29th, 2020 eTable 1. Parallel Trend Assumption Test eTable 2. Association of State STLD Plan Policies and Later-Stage Cancer Diagnoses: Sensitivity Analysis for Female Breast Cancer and Colorectal Cancer in Age Group with Recommended Asymptomatic Screening eTable 3. Association of State Short-Term Limited Duration Plan Policies and Later-Stage Cancer Diagnoses: Falsification Test Among Adults Aged 66-70 Years Age Eligible for Medicare Coverage eTable 4. Association of State STLD Plan Policies and Later-Stage Cancer Diagnoses: Sensitivity Analysis Adjusting for Zip-Code Education Attainment and State-Level Uninsured Rate eTable 5. Association of State STLD Plan Policies and Later-Stage Cancer Diagnoses With 99% Confidence Interval [file jamanetwopen-e251075-s001.pdf]

Supplemental Online Content

Yang NN, Zhao J, Barnes JM, et al. Short-term limited duration insurance plan policies and cancer stage at diagnosis. *JAMA Netw Open.* 2025;8(3):e251075. doi:10.1001/jamanetworkopen.2025.1075

eFigure 1. Study Sample

eFigure 2. STLD Policy Status by States as of February 29th, 2020

eTable 1. Parallel Trend Assumption Test

eTable 2. Association of State STLD Plan Policies and Later-Stage Cancer Diagnoses: Sensitivity Analysis for Female Breast Cancer and Colorectal Cancer in Age Group with Recommended Asymptomatic Screening

eTable 3. Association of State Short-Term Limited Duration Plan Policies and Later-Stage Cancer Diagnoses: Falsification Test Among Adults Aged 66-70 Years Age Eligible for Medicare Coverage

eTable 4. Association of State STLD Plan Policies and Later-Stage Cancer Diagnoses: Sensitivity Analysis Adjusting for Zip-Code Education Attainment and State-Level Uninsured Rate

eTable 5. Association of State STLD Plan Policies and Later-Stage Cancer Diagnoses With 99% Confidence Interval

This supplemental material has been provided by the authors to give readers additional information about their work.

eFigure 1. Study sample

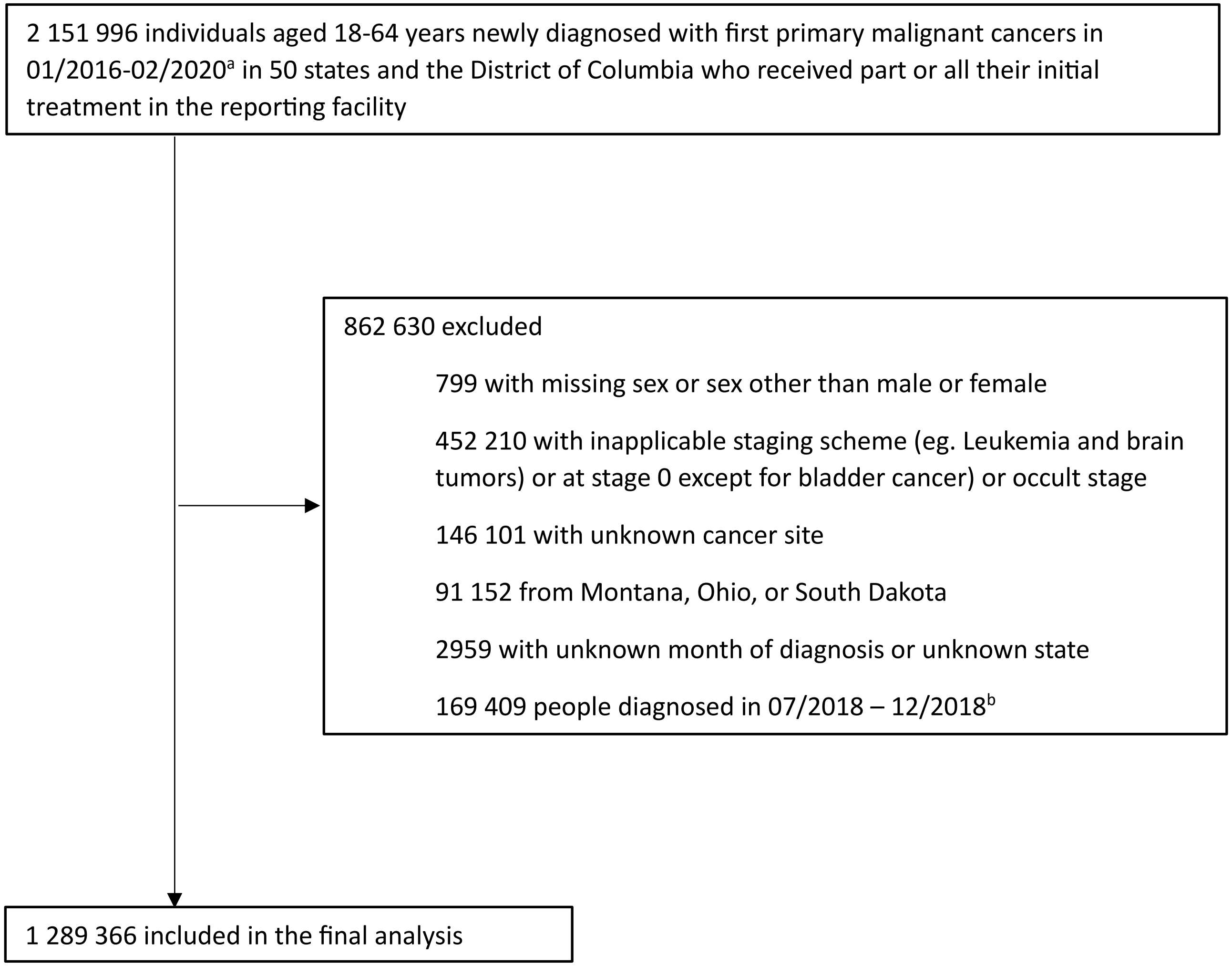

a We set the cutoff as February 2020 to avoid the disruption of cancer diagnosis by the COVID-19 outbreak in March 2020.

b Exclusion of people diagnosed during policy phase: There can be a washout period after the federal rule when states implement policies at different time points. To better study the policy of focus, we excluded cases diagnosed between July 2018 and December 2018.

© 2025 Yang NN et al. *JAMA Network Open*.

eFigure 2. STLD policy status by states as of February 29th, 2020

Group 1: New York, New Jersey, Massachusetts, Rhode Island, Vermont;

Group 2: California, Connecticut, Hawaii, New Mexico, Colorado, Maine;

Group 3: DC, Delaware, Illinois, Kansas, Maryland, Michigan, Minnesota, Missouri, North Dakota, New Hampshire, Nevada, Oregon, South Carolina, Washington, Wisconsin;

Group 4: Alabama, Alaska, Arkansas, Arizona, Florida, Georgia, Iowa, Idaho, Indiana, Kentucky, Louisiana, Mississippi, Nebraska, North Carolina, Oklahoma, Pennsylvania, Tennessee, Utah, Texas, Virginia, West Virginia, Wyoming.

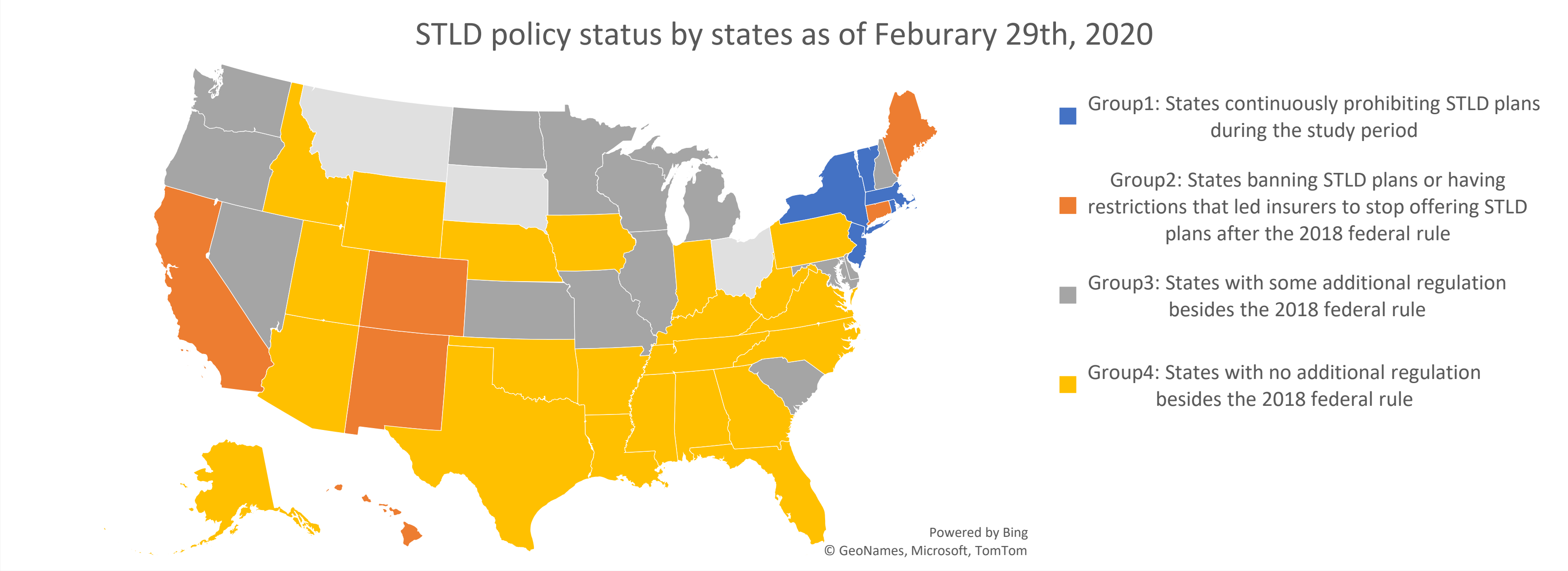

© 2025 Yang NN et al. *JAMA Network Open*.

eTable 1. Parallel trend assumption test

**Cancer type**	**P value**
All cancer	0.27
Female Breast	0.22
Prostate	0.44
Colorectal	0.94
Non-Small Cell Lung	0.46
Melanoma	0.21

Data source: National Cancer Database (NCDB) 2016-2020.
To test the parallel trends assumption that there are no differences in groups prior to policy change, we examined the expansion-by-quarter interaction terms for January 2016 to June 2018. P-values >0.05 suggest that the parallel trends assumption is not violated.

© 2025 Yang NN et al. *JAMA Network Open*.

eTable 2. Association of State STLD Plan Policies and Later-Stage Cancer Diagnoses: Sensitivity Analysis for Female Breast Cancer and Colorectal Cancer in Age Group with Recommended Asymptomatic Screening

Characteristic		2016-2018 (%)	2019-2020 (%)	Absolute difference (ppt) (95% CI)	Unadjusted Model		Adjusted Model	
					DiD (95% CI)	P	DiD (95% CI)	P
Female Breast Cancer (40-64)	Group 1	12.7	10.9	-1.8 (-2.53to-1.11)	Ref	Ref	Ref	Ref
	Group 2	14.0	12.9	-1.1 (-1.79to-0.40)	0.7 (-0.27-1.71)	0.15	0.7 (-0.29-1.68)	0.17
	Group 3	13.5	12.3	-0.8 (-1.30to-0.30)	0.6 (-0.30-1.47)	0.19	0.7 (-0.23-1.53)	0.15
	Group 4	15.0	13.9	-1.2 (-1.76to-0.71)	0.8 (-0.02-1.60)	0.06	0.8 (0.03-1.65)	0.04
Colorectal Cancer (50-64)	Group 1	53.9	56.4	-1.0 (-1.42to-0.63)	Ref	Ref	Ref	Ref
	Group 2	51.6	55.4	3.8 (1.94-5.60)	1.2 (-1.41-3.87)	0.36	1.3 (-1.31-3.96)	0.32
	Group 3	52.4	57.5	5.1 (3.76-6.47)	2.6 (0.24-4.91)	0.03	2.6 (0.29-4.95)	0.03
	Group 4	55.4	59.0	3.5 (2.54-4.51)	1.0 (-1.16-3.12)	0.37	1.0 (-1.17-3.11)	0.37

Data source: National Cancer Database (NCDB) 2016-2020.

Group 1(States continuously prohibiting STLD plans during the study period): New York, New Jersey, Massachusetts, Rhode Island, Vermont

Group 2(States banning STLD plans or having restrictions that led insurers to stop offering STLD plans after the 2018 federal rule): California, Connecticut, Hawaii, New Mexico, Colorado, Maine

Group 3(States with some additional regulation besides the 2018 federal rule): DC, Delaware, Illinois, Kansas, Maryland, Michigan, Minnesota, Missouri, North Dakota, New Hampshire, Nevada, Oregon, South Carolina, Washington, Wisconsin

Group 4(States with no additional regulation besides the 2018 federal rule): Alabama, Alaska, Arkansas, Arizona, Florida, Georgia, Iowa, Idaho, Indiana, Kentucky, Louisiana, Mississippi, Nebraska, North Carolina, Oklahoma, Pennsylvania, Tennessee, Utah, Texas, Virginia, West Virginia, Wyoming

© 2025 Yang NN et al. *JAMA Network Open*.

eTable 3. Association of State Short-Term Limited Duration Plan Policies and Later-Stage Cancer Diagnoses: Falsification Test among Adults aged 66-70 Years Age Eligible for Medicare Coverage

Characteristic		2016-2018	2019-2020	Absolute difference (ppt) (95% CI)	Unadjusted Model		Adjusted Model	
					DiD (95% CI)	P	DiD (95% CI)	P
**All cancers**	Group 1 (n=54,077)	37.2	37.7	0.5 (-0.39-1.36)	Ref	Ref	Ref	Ref
	Group 2 (n=55,024)	37.2	37.0	-0.2 (-1.08-0.65)	-0.7 (-1.93-0.53)	0.26	-0.6 (-1.79-0.65)	0.36
	Group 3 (n=110,137)	37.4	37.9	0.5 (-0.14-1.08)	0.0 (-1.08-1.05)	0.98	0.1 (-0.96-1.15)	0.86
	Group 4 (n=194,845)	38.1	38.1	0.0 (-0.45-0.47)	-0.5 (-1.46-0.51)	0.35	-0.3 (-1.25-0.7)	0.58
**Female breast**	Group 1 (n=10,021)	11.2	9.6	-1.6 (-2.92-0.31)	Ref	Ref	Ref	Ref
	Group 2 (n=10,991)	11.5	8.8	-2.6 (-3.85to-1.37)	-1 (-2.80-0.80)	0.28	-0.9 (-2.74-0.85)	0.30
	Group 3 (n=20,698)	10.7	9.3	-1.4 (-2.35to-0.54)	0.2 (-1.43-1.75)	0.84	0.2 (-1.34-1.84)	0.76
	Group 4 (n=37,230)	11.6	9.9	-1.7 (-2.304to-1.00)	-0.1 (-1.53-1.41)	0.94	0.0 (-1.47-1.47)	1.00
**Prostate**	Group 1 (n=11,731)	28.1	31.6	3.4 (1.67-5.14)	Ref	Ref	Ref	Ref
	Group 2 (n=12,149)	29.7	36.0	6.3 (4.58-7.99)	2.9 (0.44-5.31)	0.02	3.1 (0.63-5.47)	0.01
	Group 3 (n=24,654)	27.9	35.2	7.3 (6.05-8.47)	3.9 (1.74-5.96)	<.001	4.0 (1.88-6.09)	<.001
	Group 4 (n=40,165)	26.9	33.9	7.0 (6.07-7.96)	3.6 (1.65-5.58)	<.001	3.7 (1.74-5.67)	<.001
**Colorectal**	Group 1 (n=4,405)	50.4	54.1	3.7 (0.52-6.91)	Ref	Ref	Ref	Ref
	Group 2 (n=4,660)	50.2	53.2	3.1 (-0.06-6.18)	-0.6 (-5.12-3.82)	0.78	-0.7 (-5.15-3.78)	0.76
	Group 3 (n=9,060)	50.1	54.5	4.4 (2.22-6.66)	0.7 (03.16-4.62)	0.71	0.8 (-3.13-4.64)	0.70
	Group 4 (n=17,676)	50.2	51.9	1.7 (0.12-3.30)	-2.0 (-5.57-1.57)	0.27	-2.1 (-5.63-1.51)	0.26
**Non-Small Cell Lung**	Group 1 (n=7,654)	56.3	56.5	0.2 (-2.14-2.61)	Ref	Ref	Ref	Ref
	Group 2 (n=6,474)	61.3	61.5	0.2 (-2.42-2.79)	0.0 (-3.58-3.48)	0.98	-0.1 (-3.63-3.39)	0.95
	Group 3 (n=15,546)	59.5	56.6	-2.8 (-4.51to-1.17)	-3.1 (-5.98to-0.17)	0.04	-3.1 (-6to-0.23)	0.03
	Group 4 (n=28,995)	59.4	57.9	-1.6 (-2.79to-0.33)	-1.8 (-4.47-0.88)	0.19	-1.7 (-4.39-0.93)	0.20
**Melanoma**	Group 1 (n=1,689)	17.9	18.6	0.8 (-3.20-4.72)	Ref	Ref	Ref	Ref
	Group 2 (n=2,202)	17.6	15.6	-2.0 (-5.51-1.48)	-2.8 (-8.06-2.5)	0.30	-2.7 (-7.98-2.55)	0.31
	Group 3 (n=4,007)	16.7	17.1	0.5 (-2.11-3.06)	-0.3 (-5.02-4.44)	0.90	-0.3 (-5.06-4.37)	0.89
	Group 4 (n=6,795)	19.6	20.7	1.1 (-0.86-3.06)	0.3 (-4.08-4.75)	0.88	0.4 (-3.97-4.84)	0.85

Data source: National Cancer Database (NCDB) 2016-2020.

© 2025 Yang NN et al. *JAMA Network Open*.

Group 1(States continuously prohibiting STLD plans during the study period): New York, New Jersey, Massachusetts, Rhode Island, Vermont
Group 2(States banning STLD plans or having restrictions that led insurers to stop offering STLD plans after the 2018 federal rule): California, Connecticut, Hawaii, New Mexico, Colorado, Maine
Group 3(States with some additional regulation besides the 2018 federal rule): DC, Delaware, Illinois, Kansas, Maryland, Michigan, Minnesota, Missouri, North Dakota, New Hampshire, Nevada, Oregon, South Carolina, Washington, Wisconsin
Group 4(States with no additional regulation besides the 2018 federal rule): Alabama, Alaska, Arkansas, Arizona, Florida, Georgia, Iowa, Idaho, Indiana, Kentucky, Louisiana, Mississippi, Nebraska, North Carolina, Oklahoma, Pennsylvania, Tennessee, Utah, Texas, Virginia, West Virginia, Wyoming

© 2025 Yang NN et al. *JAMA Network Open*.

eTable 4. Association of State STLD Plan Policies and Later-Stage Cancer Diagnoses: Sensitivity Analysis Adjusting for Zip-Code Education Attainment and State-level Uninsured Rate

Characteristic		2016-2018	2019-2020	Absolute difference (ppt) (95% CI)	Unadjusted Model		Adjusted Model	
					DiD (95% CI)	P	DiD (95% CI)	P
All cancers	Group 1 (n=175,322)	33.8	32.1	-1.7 (-2.29to-1.03)	Ref	Ref	Ref	Ref
	Group 2 (n=174,080)	34.1	32.6	-1.4 (-2.07to-0.80)	0.2 (-0.46-0.91)	0.52	0.4 (-0.22, 1.11)	0.19
	Group 3 (n=335,082)	35.4	34.4	-1.0 (-1.5to-0.58)	0.6 (0.02-1.22)	0.04	0.9 (0.27, 1.44)	0.004
	Group 4 (n=604,882)	37.1	36.0	-1.1 (-1.42to-0.74)	0.6 (0.03-1.13)	0.04	0.8 (0.23, 1.3)	0.005
Female breast	Group 1 (n=45,866)	13.5	12.0	-1.5 (-2.39to-0.57)	Ref	Ref	Ref	Ref
	Group 2 (n=47,825)	14.8	14.1	-0.7 (-1.57-0.20)	0.8 (-0.17-1.76)	0.11	0.8 (-0.12, 1.8)	0.09
	Group 3 (n=83,909)	14.3	13.5	-0.8 (-1.5to-0.15)	0.6 (-0.21-1.51)	0.14	0.7 (-0.12, 1.6)	0.09
	Group 4 (n=150,077)	15.7	15.1	-0.6 (-1.1to-0.09)	0.9 (0.09-1.68)	0.03	1.0 (0.22, 1.8)	0.01
Prostate	Group 1 (n=23,323)	27.4	30.7	3.3 (1.69-4.93)	Ref	Ref	Ref	Ref
	Group 2 (n=19,865)	29.3	33.5	4.2 (2.44-5.96)	0.9 (-0.94-2.70)	0.34	1.0 (-0.82, 2.81)	0.28
	Group 3 (n=44,985)	27.1	33.2	6.1 (4.91-7.27)	2.8 (1.25-4.30)	<.001	2.9 (1.39, 4.43)	<.001
	Group 4 (n=75,054)	26.8	33.3	6.6 (5.65-7.47)	3.2 (1.83-4.66)	<.001	3.3 (1.89, 4.71)	<.001
Colorectal	Group 1 (n=16,573)	55.6	58.3	2.7 (0.61-4.84)	Ref	Ref	Ref	Ref
	Group 2 (n=18,753)	53.9	58.4	4.5 (2.48-6.47)	1.8 (-0.46-3.97)	0.12	1.7 (-0.53, 3.88)	0.14
	Group 3 (n=34,017)	54.4	59.8	5.3 (3.84-6.83)	2.6 (0.64-4.58)	0.01	2.6 (0.61, 4.54)	0.01
	Group 4 (n=64,453)	57.2	60.7	3.6 (2.49-4.66)	0.9 (-0.95-2.66)	0.35	0.7 (-1.07, 2.54)	0.42
Non-Small Cell Lung	Group 1 (n=15,085)	63.4	61.7	-1.7 (-3.85-0.42)	Ref	Ref	Ref	Ref
	Group 2 (n=11,331)	69.4	68.9	-0.5 (-2.97-2.01)	1.2 (-1.26-3.73)	0.33	1.2 (-1.29, 3.66)	0.35
	Group 3 (n=29,627)	67.0	65.5	-1.5 (-3.04-0.02)	0.2 (-1.79-2.21)	0.84	0.3 (-1.67, 2.29)	0.76
	Group 4 (n=56,796)	68.9	67.4	-1.4 (-2.48to-0.27)	0.3 (-1.49-2.17)	0.72	0.5 (-1.27, 2.35)	0.56
Melanoma	Group 1 (n=7,063)	17.3	17.7	0.5 (-2.20-3.12)	Ref	Ref	Ref	Ref
	Group 2 (n=8,543)	18.7	20.6	1.9 (-0.56-4.34)	1.4 (-1.32-4.18)	0.31	1.2 (-1.29, 3.66)	0.35
	Group 3 (n=17,744)	18.9	19.1	0.2 (-1.43-1.9)	-0.2 (-2.62-2.17)	0.85	0.3 (-1.67, 2.29)	0.76
	Group 4 (n=28,245)	21.1	22.1	1.1 (-0.25-2.38)	0.6 (-1.65-2.87)	0.6	0.5 (-1.27, 2.35)	0.56

Data source: National Cancer Database (NCDB) 2016-2020.

© 2025 Yang NN et al. *JAMA Network Open*.

Group 1(States continuously prohibiting STLD plans during the study period): New York, New Jersey, Massachusetts, Rhode Island, Vermont
Group 2(States banning STLD plans or having restrictions that led insurers to stop offering STLD plans after the 2018 federal rule): California, Connecticut, Hawaii, New Mexico, Colorado, Maine
Group 3(States with some additional regulation besides the 2018 federal rule): DC, Delaware, Illinois, Kansas, Maryland, Michigan, Minnesota, Missouri, North Dakota, New Hampshire, Nevada, Oregon, South Carolina, Washington, Wisconsin
Group 4(States with no additional regulation besides the 2018 federal rule): Alabama, Alaska, Arkansas, Arizona, Florida, Georgia, Iowa, Idaho, Indiana, Kentucky, Louisiana, Mississippi, Nebraska, North Carolina, Oklahoma, Pennsylvania, Tennessee, Utah, Texas, Virginia, West Virginia, Wyoming

© 2025 Yang NN et al. *JAMA Network Open*.

eTable 5. Association of State STLD Plan Policies and Later-Stage Cancer Diagnoses with 99% Confidence Interval

Characteristic		2016-2018	2019-2020	Absolute difference (ppt) (99% CI)	Unadjusted Model		Adjusted Model	
					DiD (99% CI)	P	DiD (99% CI)	P
**All cancers**	Group 1 (n=175,322)	33.8	32.1	-1.7 (-2.29to-1.03)	Ref	Ref	Ref	Ref
	Group 2 (n=174,080)	34.1	32.6	-1.4 (-2.07to-0.80)	0.2 (-0.46-0.91)	0.52	0.5 (-0.43-1.33)	0.19
	Group 3 (n=335,082)	35.4	34.4	-1.0 (-1.5to-0.58)	0.6 (0.02-1.22)	0.04	0.8 (0.07-1.60)	0.005
	Group 4 (n=604,882)	37.1	36.0	-1.1 (-1.42to-0.74)	0.6 (0.03-1.13)	0.04	0.8 (0.05-1.46)	0.01
**Female breast**	Group 1 (n=45,866)	13.5	12.0	-1.5 (-2.39to-0.57)	Ref	Ref	Ref	Ref
	Group 2 (n=47,825)	14.8	14.1	-0.7 (-1.57-0.20)	0.8 (-0.17-1.76)	0.11	0.8 (-0.43-2.09)	0.09
	Group 3 (n=83,909)	14.3	13.5	-0.8 (-1.5to-0.15)	0.6 (-0.21-1.51)	0.14	0.7 (-0.39-1.86)	0.09
	Group 4 (n=150,077)	15.7	15.1	-0.6 (-1.1to-0.09)	0.9 (0.09-1.68)	0.03	1.0 (-0.03-2.04)	0.01
**Prostate**	Group 1 (n=23,323)	27.4	30.7	3.3 (1.69-4.93)	Ref	Ref	Ref	Ref
	Group 2 (n=19,865)	29.3	33.5	4.2 (2.44-5.96)	0.9 (-0.94-2.70)	0.34	1.0 (-1.40-3.37)	0.29
	Group 3 (n=44,985)	27.1	33.2	6.1 (4.91-7.27)	2.8 (1.25-4.30)	<.001	2.9 (0.88-4.88)	<.001
	Group 4 (n=75,054)	26.8	33.3	6.6 (5.65-7.47)	3.2 (1.83-4.66)	<.001	3.3 (1.42-5.12)	<.001
**Colorectal**	Group 1 (n=16,573)	55.6	58.3	2.7 (0.61-4.84)	Ref	Ref	Ref	Ref
	Group 2 (n=18,753)	53.9	58.4	4.5 (2.48-6.47)	1.8 (-0.46-3.97)	0.12	1.7 (-1.22-4.58)	0.13
	Group 3 (n=34,017)	54.4	59.8	5.3 (3.84-6.83)	2.6 (0.64-4.58)	0.01	2.6 (-0.02-5.13)	0.01
	Group 4 (n=64,453)	57.2	60.7	3.6 (2.49-4.66)	0.9 (-0.95-2.66)	0.35	0.7 (-1.65-3.09)	0.43
**Non-Small Cell Lung**	Group 1 (n=15,085)	63.4	61.7	-1.7 (-3.85-0.42)	Ref	Ref	Ref	Ref
	Group 2 (n=11,331)	69.4	68.9	-0.5 (-2.97-2.01)	1.2 (-1.26-3.73)	0.33	1.2 (-2.10-4.40)	0.36
	Group 3 (n=29,627)	67.0	65.5	-1.5 (-3.04-0.02)	0.2 (-1.79-2.21)	0.84	0.3 (-2.29-2.91)	0.76
	Group 4 (n=56,796)	68.9	67.4	-1.4 (-2.48to-0.27)	0.3 (-1.49-2.17)	0.72	0.5 (-1.87-2.89)	0.58
**Melanoma**	Group 1 (n=7,063)	17.3	17.7	0.5 (-2.20-3.12)	Ref	Ref	Ref	Ref
	Group 2 (n=8,543)	18.7	20.6	1.9 (-0.56-4.34)	1.4 (-1.32-4.18)	0.31	1.5 (-2.12-5.04)	0.29
	Group 3 (n=17,744)	18.9	19.1	0.2 (-1.43-1.9)	-0.2 (-2.62-2.17)	0.85	-0.1 (-3.24-2.99)	0.91
	Group 4 (n=28,245)	21.1	22.1	1.1 (-0.25-2.38)	0.6 (-1.65-2.87)	0.6	0.6 (-2.39-3.50)	0.63

Data source: National Cancer Database (NCDB) 2016-2020.

© 2025 Yang NN et al. *JAMA Network Open*.

Group 1(States continuously prohibiting STLD plans during the study period): New York, New Jersey, Massachusetts, Rhode Island, Vermont
Group 2(States banning STLD plans or having restrictions that led insurers to stop offering STLD plans after the 2018 federal rule): California, Connecticut, Hawaii, New Mexico, Colorado, Maine
Group 3(States with some additional regulation besides the 2018 federal rule): DC, Delaware, Illinois, Kansas, Maryland, Michigan, Minnesota, Missouri, North Dakota, New Hampshire, Nevada, Oregon, South Carolina, Washington, Wisconsin
Group 4(States with no additional regulation besides the 2018 federal rule): Alabama, Alaska, Arkansas, Arizona, Florida, Georgia, Iowa, Idaho, Indiana, Kentucky, Louisiana, Mississippi, Nebraska, North Carolina, Oklahoma, Pennsylvania, Tennessee, Utah, Texas, Virginia, West Virginia, Wyoming

© 2025 Yang NN et al. *JAMA Network Open*.
