# Supplementary material for: Short-Term Limited Duration Insurance Plan Policies and Cancer Stage at Diagnosis
Source: JAMA Netw Open. 2025 Mar 18;8(3):e251075. doi: 10.1001/jamanetworkopen.2025.1075 (PMC11920836; doi:10.1001/jamanetworkopen.2025.1075)
Supplement: Supplement 2. — Data Sharing Statement [file jamanetwopen-e251075-s002.pdf]

Data Sharing Statement

Yang. Short-Term Limited Duration Insurance Plan Policies and Cancer Stage at Diagnosis. *JAMA Netw Open*. Published March 18, 2025. doi:10.1001/jamanetworkopen.2025.1075

Data
Data available: No

Additional Information
Explanation for why data not available: The data underlying this article were provided by the American College of Surgeons and accessed at the American Cancer Society by permission. The data cannot be shared publicly per the Data User Agreement. The National Cancer Database Participant User Files are available through application to investigators associated with the Commission on Cancer accredited cancer programs (https://www.facs.org/quality-programs/cancer/ncdb/puf).
